# Inflammatory Bowel Disease-Associated Gut Commensals Degrade Components of the Extracellular Matrix

**DOI:** 10.1128/mbio.02201-22

**Published:** 2022-11-29

**Authors:** Ana Maria Porras, Hao Zhou, Qiaojuan Shi, Xieyue Xiao, JRI Live Cell Bank, Randy Longman, Ilana Lauren Brito

**Affiliations:** a J. Crayton Pruitt Family Department of Biomedical Engineering, University of Florida, Gainesville, Florida, USA; b Meinig School of Biomedical Engineering, Cornell University, Ithaca, New York, USA; c Jill Roberts Institute for IBD Research, Weill Cornell Medicine, New York, New York, USA; University of Michigan-Ann Arbor

**Keywords:** gut microbiome, inflammatory bowel disease, extracellular matrix, host-microbe interactions, colitis, proteases

## Abstract

Extracellular matrix (ECM) remodeling has emerged as a key feature of inflammatory bowel disease (IBD), and ECM fragments have been proposed as markers of clinical disease severity. Recent studies report increased protease activity in the gut microbiota of IBD patients. Nonetheless, the relationship between gut microbiota and ECM remodeling has remained unexplored. We hypothesized that members of the human gut microbiome could degrade the host ECM and that bacteria-driven remodeling, in turn, could enhance colonic inflammation. Through a variety of *in vitro* assays, we first confirmed that multiple bacterial species found in the human gut are capable of degrading specific ECM components. Clinical stool samples obtained from ulcerative colitis patients also exhibited higher levels of proteolytic activity *in vitro*, compared to those of their healthy counterparts. Furthermore, culture supernatants from bacteria species that are capable of degrading human ECM accelerated inflammation in dextran sodium sulfate (DSS)-induced colitis. Finally, we identified several of the bacterial proteases and carbohydrate degrading enzymes (CAZymes) that are potentially responsible for ECM degradation *in vitro*. Some of these protease families and CAZymes were also found in increased abundance in a metagenomic cohort of IBD. These results demonstrate that some commensal bacteria in the gut are indeed capable of degrading components of human ECM *in vitro* and suggest that this proteolytic activity may be involved in the progression of IBD. A better understanding of the relationship between nonpathogenic gut microbes, host ECM, and inflammation could be crucial to elucidating some of the mechanisms underlying host-bacteria interactions in IBD and beyond.

## INTRODUCTION

Uncontrolled remodeling of the host extracellular matrix (ECM) is a known hallmark of inflammatory bowel disease (IBD) (1–6). The intestinal ECM consists of a combination of proteins, glycoproteins, and proteoglycans that provide not only mechanical support but also important biochemical cues for the development and homeostasis of the colon (7). For example, the basement membrane ECM beneath the mucosal epithelium helps maintain intestinal barrier integrity, and the interstitial matrix ECM supports both the structural integrity and the remodeling of the submucosa (1). While the basement membrane consists primarily of collagen IV and laminin, the interstitial ECM includes a wider variety of components, including fibrillar collagen (including types I and III), fibronectin, hyaluronic acid, elastin, and several proteoglycans (1). Increased protease activity and degradation of the ECM in the intestinal mucosa and submucosa have been reported in both ulcerative colitis (UC) and Crohn’s disease (CD) (5, 8–11). Many IBD patients also suffer from intestinal fibrosis, which involves the accumulation of ECM components, such as collagen, along the lining of the colonic epithelium (3, 12–14). Excessive ECM degradation and deposition may result in the development of fistulae and strictures, respectively, with serious clinical consequences (15–17). As a result, ECM fragments and proteases have emerged as potential markers of disease severity (4, 5, 18–20).

Recent studies in mouse models (8, 21) and clinical settings (9, 22) suggest that ECM degradation precedes inflammation in UC. Thus, dysregulated ECM production is not only a product but also a promoter of inflammation and is an active player in the pathogenesis of IBD. Several studies have reported the overexpression of ECM remodeling enzymes (matrix metalloproteases, heparanases, and elastases) in the intestinal tissue of IBD patients (10, 11, 23–27). These enzymes are secreted by host cells (e.g., fibroblasts, neutrophils, and macrophages), and they have been implicated in experimental colitis through mechanisms such as increased epithelial permeability and proinflammatory signaling loops (28–30). Nonetheless, current hypotheses that seek to explain the ECM imbalance observed in IBD do not include the potential contributions of gut microbiota to these dynamic ECM processes.

While the degradation of mucin by gut microbiota has been studied extensively (31–36), there is limited knowledge regarding the ability of commensal bacteria to degrade the components of the human ECM in the gut. Bacterial pathogens have been shown to bind and degrade the ECM to invade intestinal and other host tissues (37–40). Similarly, bacteria associated with oral microbiota dysbiosis can break down components of the basal lamina, which potentially contributes to the progression of periodontal disease (41–43). Prominent members of the gut microbiome, such as Bacteroides thetaiotaomicron (*B. theta*) and Bacteroides fragilis, are also known to express sulfatases (44, 45) and gelatinases (46, 47), respectively. Furthermore, enterotoxigenic B. fragilis, found in abundance in IBD and colorectal cancer, secretes a metalloprotease that is capable of altering the endothelial barrier integrity and inducing the secretion of inflammatory cytokines (48). However, the pathological consequences of this proteolytic activity have not been explored from the perspective of bacteria-ECM interactions.

We hypothesize that multiple members of the gut microbiome can remodel the human ECM and that bacteria-driven degradation, in turn, can enhance colonic inflammation. First, we designed a series of *in vitro* assays that uncovered the ability of multiple bacterial species that are present in the human gut to degrade various ECM components. The same assays were repeated using samples collected from healthy and UC patients. The microbiota in these clinical UC samples were more proteolytically active than those of their healthy counterparts. Finally, culture supernatants from bacteria species that are capable of degrading human ECM exacerbated inflammation in a mouse model of DSS-induced colitis. Collectively, the results presented in this study suggest that gut microbiota indeed interact with and degrade host ECM in a manner that may contribute to the progression of IBD.

## RESULTS

### Commensal members of the gut microbiome can degrade ECM components *in vitro*.

First, we performed a series of *in vitro* tests to assess the ability of commensal bacteria to degrade individual host ECM components. We selected 12 bacterial strains that are abundant in human gut microbiomes, commonly used as probiotics, and known to be mucin-degraders. Additionally, some of these species (*B. theta*, B. fragilis, and *R. gnavus*) have previously been associated with inflammation and with the progression of UC (for *B. theta* and B. fragilis) or with both UC and CD (for *R. gnavus*) (44, 48–52). These strains were cultured individually in their corresponding recommended complete growth media (Table S1). Because many ECM-degrading enzymes produced by pathogens are secreted (38, 53), we performed all assays using culture supernatant. Thus, the supernatant from the bacterial cultures was collected and used in degradation assays for ECM components abundant in either the mucosa or submucosa (1): collagen I and IV, laminin, fibronectin, chondroitin sulfate, and hyaluronic acid.

10.1128/mbio.02201-22.1 TABLE S1 List of bacterial strains tested in degradation assays *in vitro*. Download Table S1, DOCX file, 0.01 MB. Copyright © 2022 Porras et al. 2022 Porras et al. https://creativecommons.org/licenses/by/4.0/ This content is distributed under the terms of the Creative Commons Attribution 4.0 International license.

**FIG 1:**
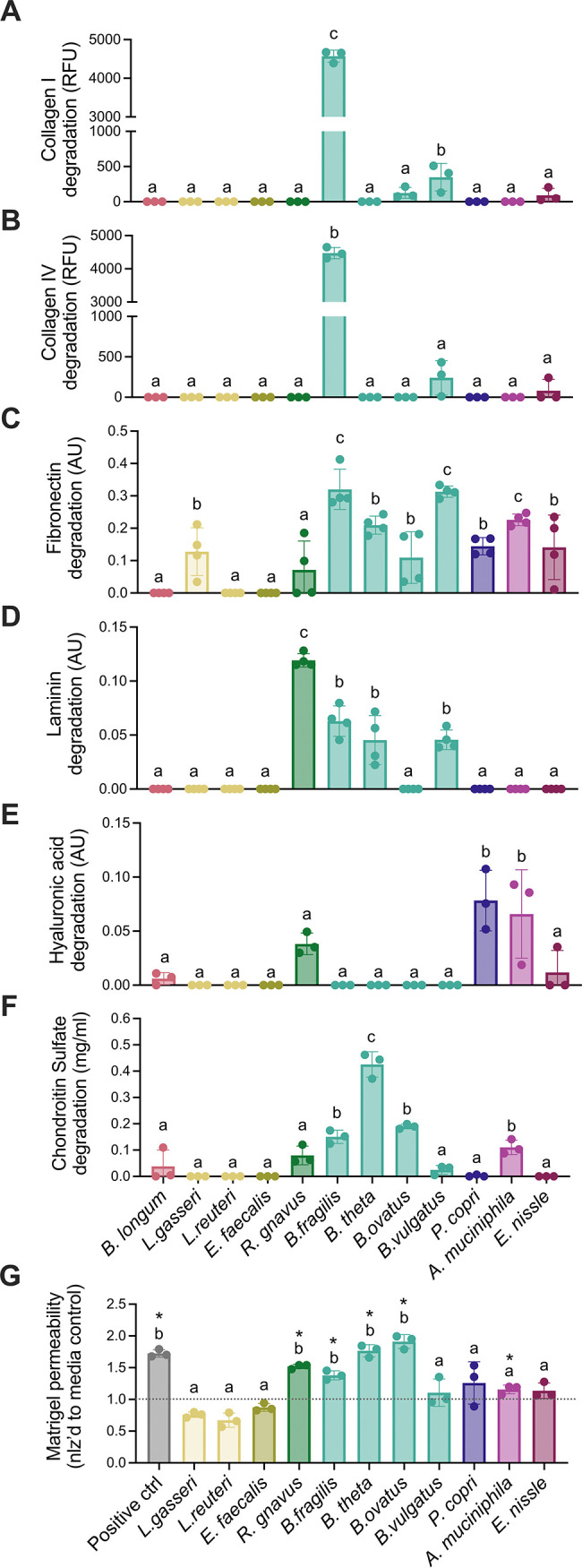
Commensal members of the gut microbiome can degrade extracellular matrix (ECM) components *in vitro*. (A–F) *In vitro* degradation of (A) collagen I, (B) collagen IV, (C) fibronectin, (D) laminin, (E) hyaluronic acid, and (F) chondroitin sulfate by the supernatant obtained from the individual culture of 12 bacterial species present in the human gut microbiome. Species represented with the same color belong to the same phylum. (G) Permeability of a Matrigel-based, *in vitro* model of the basement membrane after 24 h of culture with the bacterial supernatant. For all panels, *n* = 3 to 4 (replicated), and the data are presented as the mean ± standard deviation (SD). Same letters denote groups that are not statistically different, and different letters indicate groups that are statistically different from each other (*P* < 0.05) by a one-way analysis of variance (ANOVA) followed by Tukey’s multiple-comparison test.

In these *in vitro* assays, all ECM components were degraded by components in the supernatants of at least one species (Fig. 1A–F). B. fragilis was the primary degrader of collagen I and IV, with just one other species (Bacteroides vulgatus) exhibiting mild proteolytic activity against these proteins (Fig. 1A–B). In contrast, the remaining components were each degraded by supernatant from at least 3 different species (Fig. 1C–F). The supernatants from a few species, including *R. gnavus*, B. fragilis, and *B. theta*, were particularly active in these *in vitro* degradation tests. Additionally, supernatant obtained from the genus *Bacteroides* degraded all of the components, except for hyaluronic acid. In contrast, we detected little to no proteolytic activity in species often proposed as probiotics (54, 55), such as Lactobacillus gasseri, Lactobacillus reuteri, and Bifidobacterium longum (Fig. 1A–F).

We then developed a Matrigel-based model of the basement membrane to test ECM degradation using a more complex substrate. Bacterial culture supernatant supplemented with fluorescein isothiocyanate (FITC)-labeled dextran was added to the top of a trans-well insert that was precoated with a Matrigel layer. The Matrigel permeability after 24 h of incubation was then assessed via the measurement of fluorescence at the bottom of the well. As observed in the other *in vitro* assays, incubation with the supernatant from *R. gnavus* and bacteria from the genus *Bacteroides* (B. fragilis, *B. theta*, and B. ovatus) led to significantly higher permeability compared to media-only controls (Fig. 1G). This was not surprising, considering that most of these species had previously exhibited proteolytic activity against collagen IV and laminin, two of the most abundant components of Matrigel and the basement membrane.

**FIG 2:**
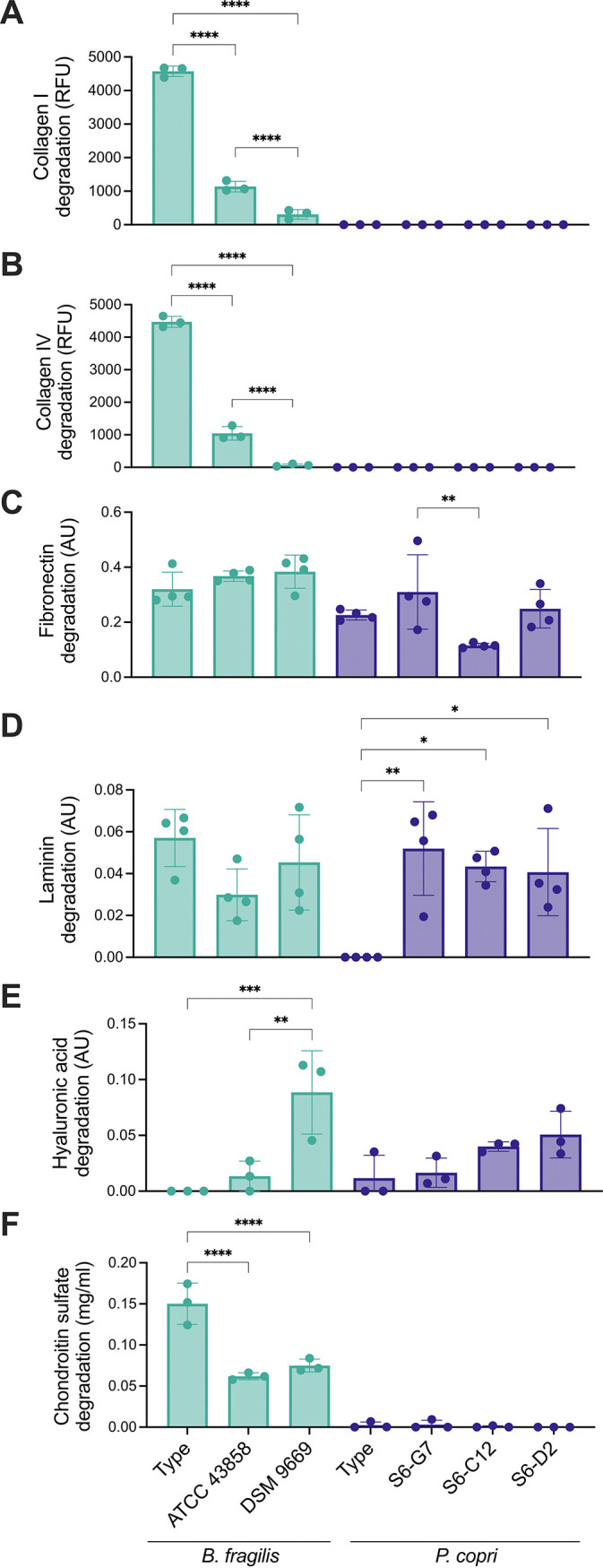
Strains and isolates of the same bacterial species exhibit differences in ECM degradation *in vitro*. (A–F) *In vitro* degradation of (A) collagen I, (B) collagen IV, (C) fibronectin, (D) laminin, (E) hyaluronic acid, and (F) chondroitin sulfate by the supernatant from the B. fragilis strains and P. copri isolates. Bars of the same color indicate the same species. For all panels, *n* = 3 to 4 (replicated), and the data are presented as the mean ±SD. *, *P* < 0.05; **, *P* < 0.01; ***, *P* < 0.001; and ****, *P* < 0.0001 by a one-way ANOVA followed by Tukey’s multiple-comparison test.

To complement our findings, we also evaluated strain-level and isolate-level differences using the same *in vitro* assays (Fig. 2). We selected two additional clinical specimens of B. fragilis strains (ATCC 43858 and DSM 9669) for comparison against the type strain (ATCC 25285) (Table S1). Additionally, we included three Prevotella copri isolates obtained from a participant in the FijiCOMP project (56) for comparison against the type strain (DSM 18205). For most of the ECM components evaluated, there were statistically significant differences between additional strains, and isolates, and the corresponding type strain (Fig. 2). For example, the B. fragilis type strain degraded collagen I, collagen IV, and chondroitin sulfate to a greater extent than did either of the other strains (Fig. 2A–B, F). In the case of laminin (Fig. 2D) and hyaluronic acid (Fig. 2E), the *P. copri* and B. fragilis type strains, respectively, exhibited no enzymatic activity, whereas the isolates and the commensal strains were indeed capable of breaking down these components. These results highlight the importance of considering strain-level differences in microbiome studies.

### Supernatant from clinical ulcerative colitis samples exhibits higher proteolytic activity.

Next, we assessed the capacity of stool community supernatants to degrade ECM components in a clinical context using the *in vitro* assays described above. We evaluated microbiota samples from healthy (*n* = 10) and UC (*n* = 9) patients (Table S2). These samples were inoculated at 2% (vol/vol) in two culture media (supplemented brain heart infusion broth [BHIS] or gut microbiome medium [GMM]) (57). Supernatants from these cultures were collected 24 h after inoculation and subjected to the same *in vitro* single-component ECM degradation assays described above.

10.1128/mbio.02201-22.2 TABLE S2 Metadata of healthy and UC patients participating in this study. Download Table S2, DOCX file, 0.01 MB. Copyright © 2022 Porras et al. 2022 Porras et al. https://creativecommons.org/licenses/by/4.0/ This content is distributed under the terms of the Creative Commons Attribution 4.0 International license.

**FIG 3:**
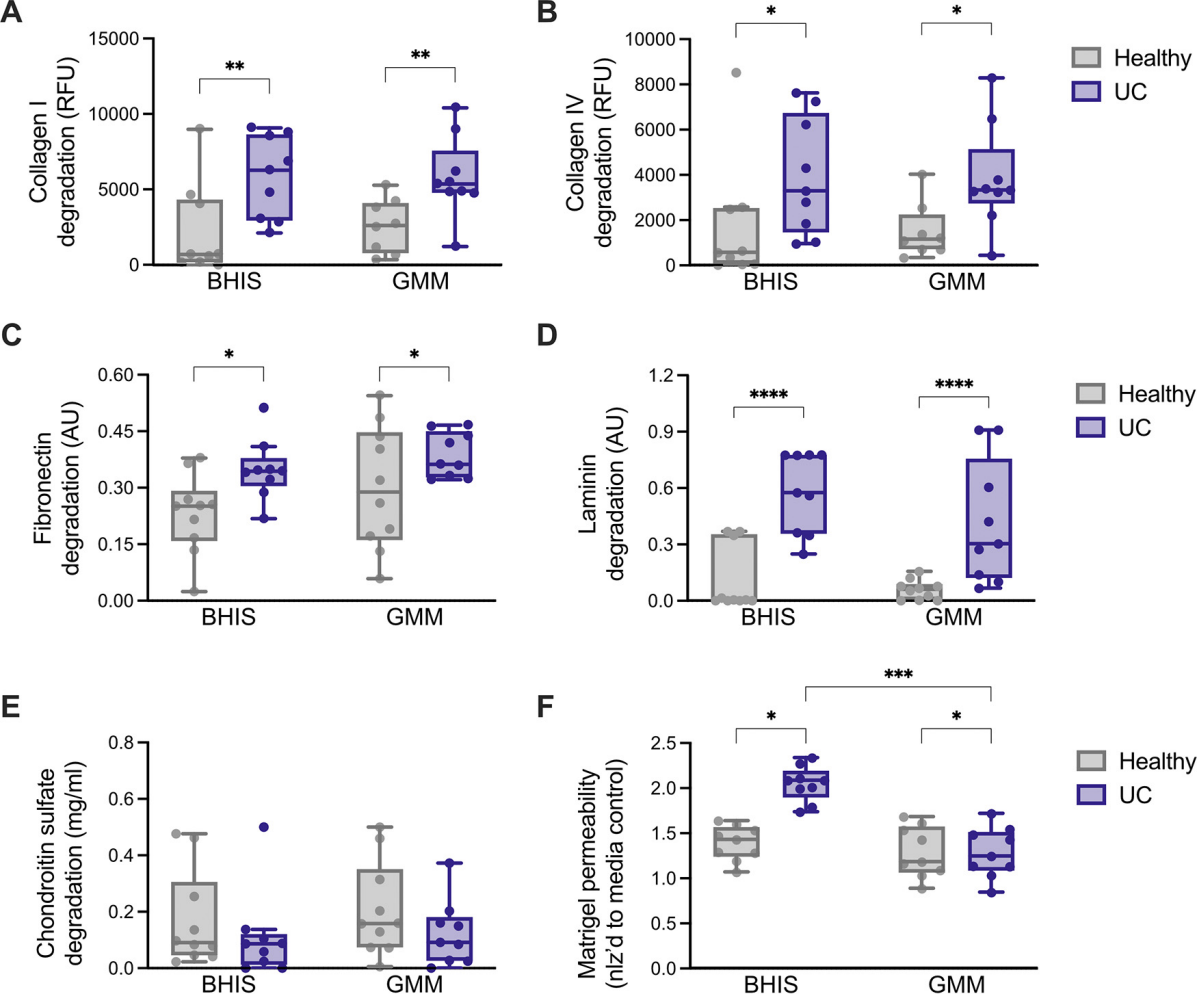
The supernatant from clinical ulcerative colitis samples exhibits higher proteolytic activity. Samples obtained from UC and healthy patients were cultured for 24 h in brain heart infusion broth (BHIS) or gut microbiome medium (GMM). Culture supernatant was then subjected to a variety of ECM degradation assays. (A to E) *In vitro* degradation of (A) collagen I, (B) collagen IV, (C) fibronectin, (D) laminin, and (E) chondroitin sulfate by the supernatant from UC and healthy patient microbiota cultures. (F) Permeability of a Matrigel-based *in vitro* model of the basement membrane after 24 h of culture with the supernatant from UC and healthy patient microbiota cultures. For all panels, *n* = 9 to 10, and the data are presented as the mean ±SD. *, *P* < 0.05; **, *P* < 0.01; ***, *P* < 0.001; and ****, *P* < 0.0001 by a one-way ANOVA followed by Tukey’s multiple-comparison test.

In general, the supernatants obtained from UC patients were better able to degrade individual ECM substrates, compared to their healthy counterparts (Fig. 3). More specifically, the UC samples exhibited increased proteolytic activity against collagen I and IV (Fig. 3A–B), fibronectin (Fig. 3C), and laminin (Fig. 3D). No statistically significant differences in chondroitin sulfate degradation were observed (Fig. 3E). Similarly, no statistically significant differences in the degradation of individual ECM components were observed when comparing the BHIS and GMM growth conditions for each patient group (Fig. 3A–E). We also evaluated Matrigel permeability in the basement membrane model following incubation with patient supernatant for 12 h. As expected from previous results, incubation with UC supernatant led to higher permeability, compared to healthy supernatant, in the BHIS condition (Fig. 3F). In this case, we did observe statistically significant differences between the BHIS and GMM growth conditions in the UC group. This is perhaps driven by the observation that Matrigel permeability in the GMM controls was higher than that in the BHIS samples. Future experiments will need to further investigate and account for these differences.

We performed 16S rRNA sequencing on the patient microbiomes that were cultured in BHIS and in GMM in order to test their degradative qualities (Fig. S1). Although there were compositional differences between microbiomes grown in BHIS and GMM, the average Bray-Curtis difference was smaller between individuals’ samples that were grown in the two conditions than between individuals’ samples that were grown in the same medium (Fig. S1), suggesting that any bias due to the choice of medium preserved the identity of the sample. Despite the ability of the cultured microbiomes to degrade ECM components and Matrigel, we were only able to detect 3 species in the cultured microbiomes: B. fragilis (5 healthy; 5 UC), A. muciniphila (1 healthy; 1 UC) and *R. gnavus* (1 UC). Despite the degradative qualities of B. fragilis, its abundances after culture were higher, overall, in the healthy samples (Fig. S1). This highlights the likelihood that the degradative traits are common across a broader subset of species.

10.1128/mbio.02201-22.6 FIG S1 Individuals’ microbiome compositions retain similarities across media. (A) Relative abundance calculations for individuals’ microbiome samples after culture in BHIS medium or GMM. (B) A principal coordinates analysis (PCoA) of the samples’ species abundances. The samples are color coded according to the individual donor. (C) Same as panel B, but colored according to media. (D) Bray-Curtis distances calculated for each individual, between individuals’ samples, and for the same medium or different media. (E) Relative abundances for B. fragilis, R. gnavus, and A. muciniphila. Download FIG S1, EPS file, 2.1 MB. Copyright © 2022 Porras et al. 2022 Porras et al. https://creativecommons.org/licenses/by/4.0/ This content is distributed under the terms of the Creative Commons Attribution 4.0 International license.

### Exposure to proteolytic supernatants accelerates inflammation in a DSS-induced mouse model of IBD.

**FIG 4:**
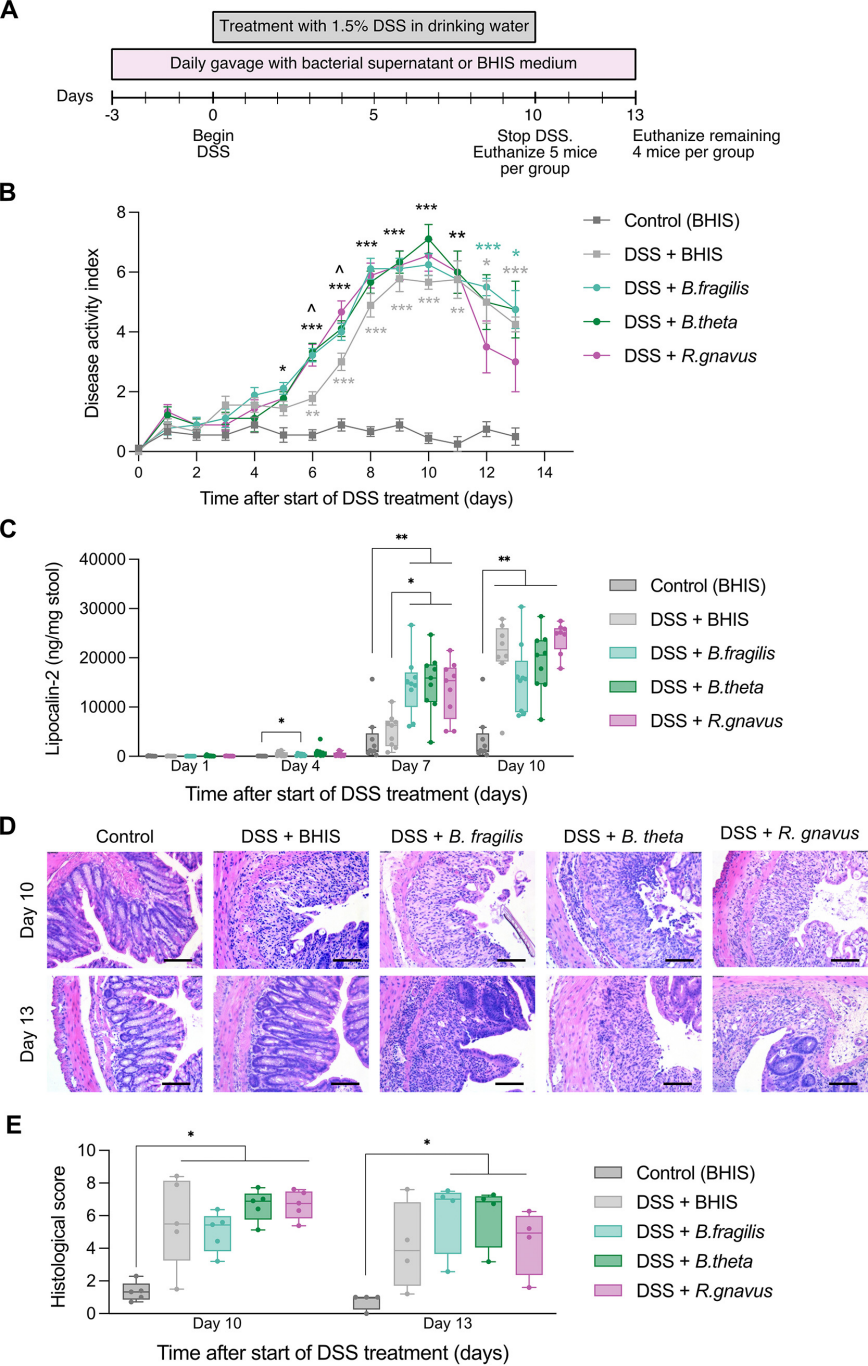
Exposure to proteolytic supernatants accelerates inflammation in a dextran sodium sulfate (DSS)-induced mouse model of inflammatory bowel disease (IBD). (A) Schematic of the *in vivo* experimental set up (*n* = 9 per experimental group). (B) Disease activity index over time after the start of DSS treatment. Data represent the mean ± SD. *, *P* < 0.05; **, *P* < 0.01; and ***, *P* < 0.001 compared to the control (BHIS). ^*P* < 0.05 compared to DSS + BHIS. Asterisks in black indicate that all of the supernatant experimental groups achieved that level of significance. (C) Quantification of lipocalin-2 levels in mouse stool at days 1, 4, 7, and 10, post-DSS treatment. *, *P* < 0.05 and **, *P* < 0.01 for the comparisons shown. (D) Hematoxylin and eosin (H&E)-stained cross-sections of explanted mouse colons on days 10 and 13. Each scale bar represents 100 μm. (E) Histological score quantifying the colonic tissue damage observed in (D). *, *P* < 0.05 for comparisons shown. For all panels, statistical significance was assessed using a mixed-effects model with the Geisser-Greenhouse correction followed by Tukey’s multiple-comparison test.

We also explored the effects of repeated exposure to bacterial supernatants in a dextran sulfate sodium salt (DSS)-induced colitis mouse model. Specifically, we selected supernatants from three of the most proteolytically active species in the *in vitro* assays: B. fragilis (ATCC 43858), *B. theta*, and *R. gnavus.* C57BL/6 mice were treated with 1.5% DSS in drinking water for 10 consecutive days to induce acute colitis. The mice were gavaged daily with either bacterial supernatant or culture medium before, during, and after DSS (Fig. 4A) (*n* = 9 mice per treatment group). Weight loss in all of the DSS-treated groups, regardless of exposure to supernatants, became significant by day 8 post-DSS treatment, peaking around 15% weight loss by day 10, with no statistically significant differences between groups (Fig. S2).

10.1128/mbio.02201-22.7 FIG S2 No significant weight loss differences were observed between treatment groups in a DSS-induced mouse model of IBD. Weight loss percentage after the start of the DSS treatment. The data represent the mean ± SD. *, *P* < 0.05; **, *P* < 0.01; and ***, *P* < 0.001 for all of the experimental groups compared to the control (BHIS). Statistical significance was assessed using a mixed-effects model with the Geisser-Greenhouse correction followed by Tukey’s multiple-comparison test. Download FIG S2, TIF file, 0.6 MB. Copyright © 2022 Porras et al. 2022 Porras et al. https://creativecommons.org/licenses/by/4.0/ This content is distributed under the terms of the Creative Commons Attribution 4.0 International license.

There were, however, differences in the timing of the onset of symptoms. Analysis of the disease activity index (DAI) curve showed equally severe clinical symptoms between the DSS-only (also treated with BHIS media as a control) and the supernatant-treated groups by day 8 post DSS-treatment (Fig. 4B). However, all of the supernatant-treated mice began exhibiting clinical signs of colitis earlier than did the DSS-only group (on day 5 rather than on day 6). Additionally, the DAI also increased more rapidly in the supernatant-treated mice between days 5 and 8. The amount of lipocalin-2 (LCN-2) in the stool, a clinical biomarker of inflammatory diseases (58), was significantly elevated in all of the DSS + supernatant-treated mice by day 7, compared to the control and DSS-only groups (Fig. 4C). By day 10, the LCN-2 levels were elevated in all of the groups that received the DSS treatment.

Mice treated with supernatant were also slower to recover than were the DSS-only treated mice. We noticed that by day 10, all of the DSS-treated groups exhibited extensive epithelial damage, crypt ablation, and mucosal erosion, as observed by histology, compared to the untreated control group (Fig. 4D–E). Greater variability in the extent of tissue damage was observed in the DSS-only group, compared to all of the other experimental groups. However, on day 13, 3 days after ending the DSS treatment, the colons of the DSS-only mice showed some evidence of recovery and improved tissue architecture (Fig. 4D). In contrast, the mice that continued to receive the daily gavage with B. fragilis, *B. theta*, and *R. gnavus* supernatants sustained statistically significant tissue damage, crypt destruction, and immune cell infiltration (Fig. 4D–E). Overall, these results suggest that a daily gavage with a proteolytically active supernatant may accelerate inflammation and sustain tissue damage *in vivo*.

### General proteases are identified in bacterial culture supernatants and are overexpressed in an IBD clinical cohort.

Finally, we sought to identify the proteases and carbohydrate degrading enzymes (CAZymes) found in each strain’s supernatant that could be responsible for the degradation of the ECM components analyzed. We performed an untargeted proteomic analysis of culture supernatants obtained from the species that exhibited significant proteolytic behavior against any of the assayed ECM components. These strains included *A. muciniphila*, B. fragilis (type, ATCC 43858, and DSM 9669), *B. ovatus*, *B. theta*, B. vulgatus, *P. copri* (Type, S6-G7, S6-C12, and S6-D2), and *R. gnavus.* After annotating the protein families (Pfams) and CAZymes secreted by each strain, we identified those previously reported to play a role in either general ECM degradation or in the degradation of specific ECM components (e.g., hyaluronic acid or laminin). These curated protein families included multiple metalloproteases (M18 and M12B) as well as several general proteases that are known to degrade a variety of ECM components, such as trypsin, papain, and calpain (Table S3). We also identified multiple CAZymes that are involved in the degradation of proteoglycans and glycosaminoglycans, such as alpha-amylase, beta-xylosidase, and both alpha- and beta-mannosidases (Table S4).

10.1128/mbio.02201-22.3 TABLE S3 Protein families (Pfams) associated with ECM degradation secreted by bacterial strains *in vitro*. List of Pfams secreted by bacterial species *in vitro* with reported roles involved in the degradation of ECM components. The “ECM Component” column indicates that Pfam was identified in the supernatant of bacteria capable of degrading that particular component. Download Table S3, DOCX file, 0.01 MB. Copyright © 2022 Porras et al. 2022 Porras et al. https://creativecommons.org/licenses/by/4.0/ This content is distributed under the terms of the Creative Commons Attribution 4.0 International license.

10.1128/mbio.02201-22.4 TABLE S4 CAZymes associated with glycosaminoglycan degradation secreted by bacterial strains *in vitro*. List of CAZymes secreted by bacterial species *in vitro* with reported roles involved in the degradation of glycosaminoglycans (in this case, HA and CS). Download Table S4, DOCX file, 0.01 MB. Copyright © 2022 Porras et al. 2022 Porras et al. https://creativecommons.org/licenses/by/4.0/ This content is distributed under the terms of the Creative Commons Attribution 4.0 International license.

**FIG 5:**
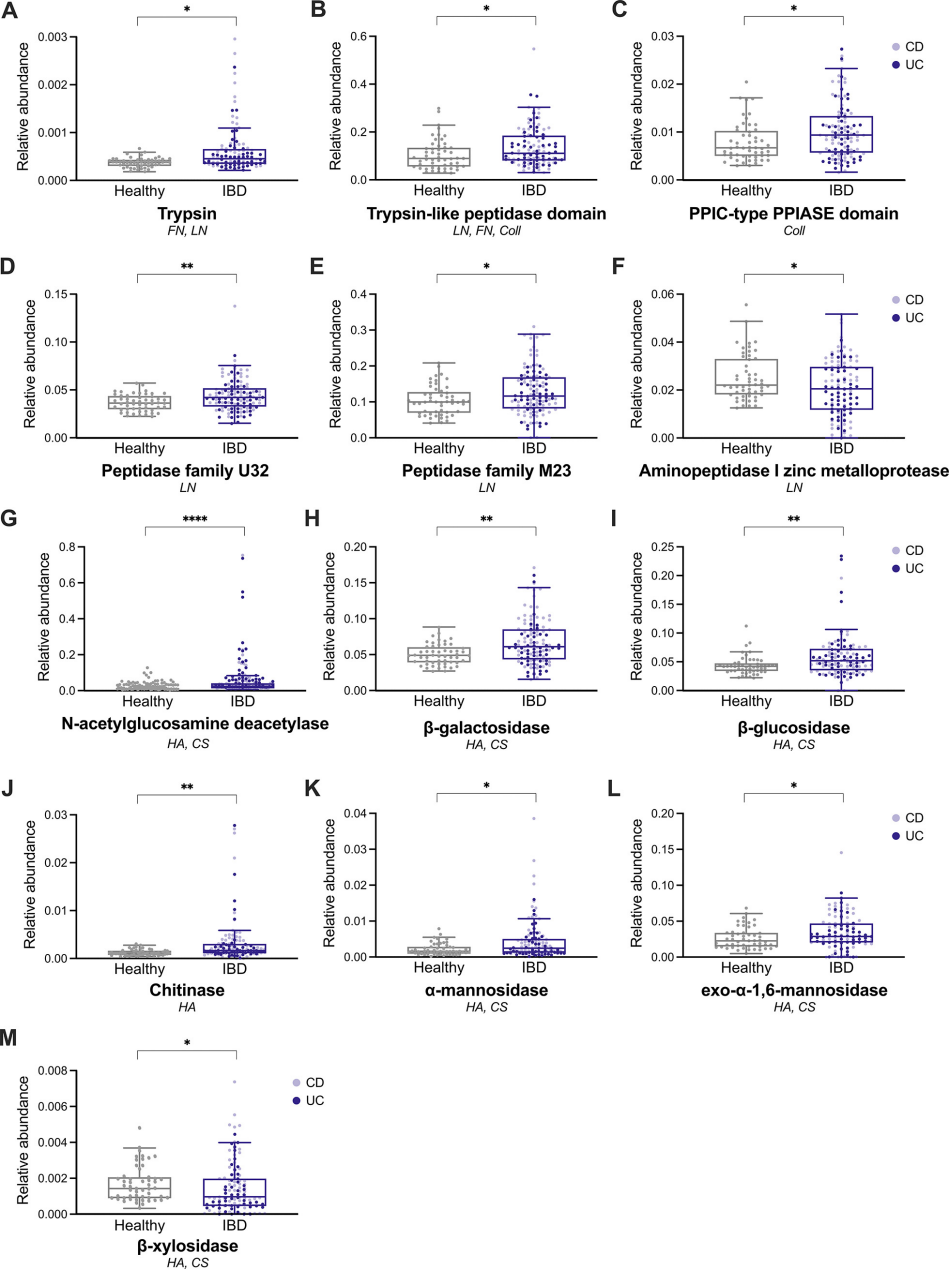
Proteases and carbohydrate degrading enzymes (CAZymes) secreted by ECM-degrading bacterial strains *in vitro* are differentially abundant in an IBD cohort compared to those of healthy controls. Relative abundance of the protein families (A–F) and CAZymes (G–M) that were found to be significantly different between IBD and healthy metagenomes from the PRISM data set (59). For each protein and enzyme family, the substrates degraded by the strain supernatants in which they were detected *in vitro* are listed. FN, fibronectin; Coll, collagen I and IV; LN, laminin; HA, hyaluronic acid; CS, chondroitin sulfate. For all panels, statistical significance was calculated using a Mann-Whitney U-test with the false discovery rate correction, and *, *P* < 0.05; **, *P* < 0.01; and ***, *P* < 0.001.

To confirm the clinical relevance of these enzymes, we assessed their relative abundances in the Prospective Registry in IBD study at MGH (PRISM) data set, a cohort that includes healthy, UC, and Crohn’s disease patients (59). 13 out of the 47 enzymes on the curated list were differentially abundant between healthy and IBD patients (Fig. 5). Among the Pfams, trypsin, trypsin-like peptidase domain, peptidyl-prolyl cis-trans isomerase (PPIC)-type PPIASE domain, and peptidase families M23 and U32 were found at a higher relative abundance in the IBD microbiomes, compared to those of the healthy controls (Fig. 5A–E). Similarly, *N*-acetylglucosamine deacetylase, β-galactosidase, β-glucosidase, chitinase, and α-mannosidase (Fig. 5G–L) were also more abundant in the IBD samples. In contrast, both the M18 zinc metalloprotease (Fig. 5F) and β-xylosidase (Fig. 5M) were relatively less abundant in IBD, compared to healthy microbiomes. These results indicate that bacteria-secreted proteases and CAZymes may be involved in the progression of IBD through the degradation of host ECM.

## DISCUSSION

ECM remodeling is increasingly recognized as a key step in the progression of disease and is a potential therapeutic target for IBD (9, 60, 61). Mounting evidence points to the increased activity of fecal (19, 22, 62) and, specifically, bacterial proteases (63, 64) associated with disease severity in UC. While these studies link bacterial proteolytic activity to inflammation, the specific mechanisms involved have not been identified. Here, we demonstrate that commensal gut microbiota secrete proteases and CAZymes capable of ECM degradation *in vitro.* Several commensal bacteria were particularly good ECM degraders, including several species of the genus *Bacteroides*, *R. gnavus*, and *P. copri.* In some cases, specific strains of *R. gnavus and* B. fragilis contribute unevenly to IBD pathophysiology (65, 66). We extend these observations, showing differences between the ECM degradation capabilities of strains of B. fragilis and *P. copri.*

We specifically identified serine and cysteine proteases, metalloproteinases, and glycosyl hydrolases, in the bacterial supernatants that were exhibiting the highest proteolytic activity *in vitro*. Of these, we found trypsin as well as several metalloproteases (peptidase families U32 and M23 as well as aminopeptidase I zinc) in increased abundance in a large metagenomic IBD cohort that included both UC and CD patients. Elevated serine and trypsin-like protease activity has also been reported in other analyses of UC and CD fecal samples (62, 63, 67), with increasing evidence that these enzymes are secreted by commensal bacteria of the *Bacteroides* genus (22, 63, 68). Similarly, zinc-dependent metalloproteases secreted by pathogenic bacteria contribute to the deterioration of intestinal barrier function through a variety of mechanisms that primarily target endothelial cells (69). Our results point to a role for these proteases that are secreted by gut commensals in the degradation of multiple ECM proteins, including collagen, laminin, and fibronectin. They also highlight the importance of CAZymes, such as α-mannosidase, β-galactosidase, and β-glucosidase, not only in the digestion of food and mucin (70) but also in the breakdown of glycosaminoglycans and glycoproteins that are commonly found in the gut ECM. Moreover, we observed similar bacterial protease and CAZyme expression levels in UC and CD patients in the metagenomic cohort (Fig. 5). Similarly, Zhihua et al. reported no differences in fecal serine protease activity between UC and CD samples (62). Thus, while our study did not include CD samples in the *in vitro* degradation assays, these results suggest that the proteolytic profile of CD microbiota may be similar to that of UC.

The secretion of these enzymes by commensals is unlikely to induce IBD on its own. In a healthy gut, the gut microbiota is confined to the intestinal lumen by a thick layer of mucus and would therefore not have access to the underlying ECM (71). In contrast, in IBD, a variety of genetic and environmental factors can disrupt the balance between the mucosal barrier and the gut microbiota. Steck et al. demonstrated that the matrix metalloprotease gelatinase E, secreted by E. faecalis, can degrade E-cadherin and can induce inflammation in a disease susceptible *IL-10^−/−^* mouse background but not in wild-type mice (72). Furthermore, disruption of the endothelial membrane in UC and CD patients leads to the invasion of colonic tissue by bacteria, including those of the genus *Bacteroides* (73, 74). Our data suggest that alterations of intestinal homeostasis could provide an opportunity for commensal-derived proteases to encounter host ECM and induce tissue damage.

ECM degradation by commensal microbiota can lead to serious consequences for the host. In this study, exposure to proteolytic supernatant in a DSS-induced model of colitis accelerated the manifestation of inflammation symptoms and led to an increase in lipocalin-2 levels. Shimshoni et al. recently demonstrated that ECM degradation precedes symptoms of inflammation in a similar mouse model (21). There are a variety of mechanisms through which bacteria-driven ECM remodeling can contribute to the progression of IBD. First, the degradation of the components of the basement membrane, such as collagen IV and laminin, could further disrupt epithelial integrity (5, 75). Second, the degradation of submucosal ECM can precipitate the recruitment and the activation of immune cells (6, 60). For example, the cleavage of hyaluronic acid (76, 77) and collagen (8) triggers the recruitment of nearby leukocytes.

Bacteria-driven ECM remodeling could also contribute not only to the pathogenesis of IBD but also to the formation of chronic fistulas and fibrostenotic lesions that could significantly impact a patient’s quality of life. Fistulas results from the excessive degradation of the interstitial ECM, which lead to transmural openings in the intestine. Increased host MMP activity and collagen type I and III degradation have been associated with fistulizing CD (4, 78, 79). Bacterial enzymes that breakdown collagen and other interstitial ECM components may also be involved in this process. While counterintuitive, bacterial ECM degradation may also be involved in the formation of fibrotic lesions through signaling cascades that ultimately lead to fibroblast activation and increased ECM deposition. The proteases could be involved in the activation of transforming growth factor-β (14), a canonical fibrotic cytokine, or in the release of other profibrotic growth factors from the interstitial ECM (3). Additionally, ECM fragments that are released after degradation can recruit and activate macrophages and other immune cells (8, 80) that, in turn, could secrete profibrotic cytokines. Further research will be necessary to begin to understand the consequences of bacteria-driven ECM degradation in the larger context of intestinal tissue remodeling.

Our results identify a potential role for gut microbiota in host ECM remodeling, and, as a result, in IBD progression. Microbiome-sourced proteases and CAZymes may serve as potential drug targets to ameliorate damage to the ECM in IBD. Sela-Paswell et al. previously demonstrated that neutralizing antibodies to host gelatinase B could be highly effective in alleviating colitis in murine models (81), with a humanized version of this antibody having recently undergone phase I clinical trials (ClinicalTrials.gov identifier: NCT01831427). Treatments alternative to immunosuppression could improve the ability to treat long-term complications that result from tissue damage. However, additional work is necessary to determine the potential success of treatments that inhibit bacterial proteases. It will be necessary to determine the relative contributions of host-derived and microbe-derived metalloproteases and to determine the specificity of each bacterial enzyme in order to predict their effects on the host. Additionally, larger cohorts will be necessary to establish whether the abundance or the total activity of these enzymes correlates with disease severity and, more importantly, ECM-associated phenotypes and disease burden (e.g., fibrotic lesions). If these enzymes are indeed correlated with disease severity, the screening of donors for ECM proteolytic activity may be an attractive strategy by which to improve the efficacy of fecal microbiota transplantation for the treatment of IBD (82). Finally, it remains to be determined how secreted bacterial enzymes gain access to the extracellular matrix, preceding overt damage to the epithelial cell lining. Nevertheless, our work provides an additional mechanistic understanding of the roles that IBD-associated bacteria play in this disease.

## MATERIALS AND METHODS

### Bacterial culture.

Bacterial strains were grown anaerobically at 37°C in an anaerobic chamber (COY Lab Products) in their corresponding complete growth medium, as outlined in Table S1. *P. copri* strains were isolated from samples collected as part of the Fiji Community Microbiome Project (FijiCOMP) (47). This study was initially approved by the Institutional Review Boards at Columbia University, the Massachusetts Institute of Technology, and the Broad Institute, and ethical approvals were received from the Research Ethics Review Committees at the Fiji National University and the Ministry of Health in the Fiji Islands. Additionally, the Cornell University Institute Review Board approved this study (number 1608006528). The human subjects provided consent prior to their participation in the study. To prepare the supernatants, liquid cultures were inoculated from frozen glycerol stocks and grown to an OD_600_ of 1.0 to 1.1. At that point, the cultures were centrifuged at 7,000 × *g* for 10 min, and the supernatant was collected and separated from the bacterial pellets. The supernatants were then refrigerated at 4°C for a maximum of 6 h until all of the supernatants were ready to begin the ECM degradation assays.

### Quantification of ECM degradation *in vitro*.

Specific degradation tests were selected for each ECM component. In all cases, the background degradation levels were considered, based on the corresponding culture medium for each bacterial supernatant. A SpectraMax (Molecular Devices) plate reader was used to measure the fluorescence and absorbance for all of the assays.

Gelatin and collagen degradation were quantified using an EnzChek Gelatinase/Collagenase Assay Kit (Thermo Fisher). DQ gelatin, collagen I, or collagen IV was added to the bacterial supernatants and the media controls at a final concentration of 50 μg/mL. Fluorescence (absorbance 495 nm, emission 515 nm) was measured in the solution following overnight incubation at 37°C under anaerobic conditions. In this case, the fluorescence was directly proportional to the gelatin and collagen degradation.

Fibronectin and laminin degradation were instead evaluated using a modified ELISA protocol that was adapted from work by Mendes et al. (83). High binding 96-well plates were coated with recombinant fibronectin (2 μg/mL; Millipore Sigma) and laminin (1 μg/mL; Millipore Sigma) diluted in PBS and were incubated overnight at 37°C. The next day, the plates were washed 3 times with 1× PBS and blocked with 3% BSA in PBS-T for at least 2 h at 37°C. After removing the BSA, bacterial supernatant was added to the corresponding wells in quadruplicate and to the plate. After a second anaerobic overnight incubation at 37°C, the plates were washed 3 times with 1× PBS to remove the supernatant. The degradation of the precoated ECM components was detected using mouse anti-fibronectin (F7387, 1:5,000; Millipore Sigma) and rabbit anti-laminin (L9393, 1:10,000; Millipore Sigma) antibodies diluted in PBS-T for 1 h at 37°C. Following another series of washes, HRP-conjugated goat anti-rabbit IgG (1:5,000; Millipore Sigma) and anti-mouse IgG (L9393, 1:5,000; Millipore Sigma) were added to the plates for 1 h at 37°C. Finally, TMB-ELISA substrate solution (Thermo Fisher) was added, and the reaction was stopped with 2N H_2_SO_4_. In this case, absorbance was inversely proportional to protein degradation.

HA degradation was analyzed in a similar fashion (84). High binding 96-well plates were coated with 200 μg/mL HA (Millipore Sigma) diluted in 0.2 M carbonate buffer (pH 9.2) and incubated overnight at 4°C. Following washing with 1× PBS, nonspecific binding was blocked with 3% BSA in PBS-T for at least 2 h at 37°C. The plates were washed, and supernatant was added to the corresponding wells and incubated anaerobically overnight at 37°C. The HA remaining after supernatant-driven degradation was detected with HA binding protein, following the dilutions and instructions in the Hyaluronan DuoSet ELISA Kit by R&D Systems (DY3614). As was the case for fibronectin and laminin, the absorbance levels were inversely proportional to the protein degradation.

The degradation of chondroitin sulfate (CS) was evaluated using a quantitative Alcian Blue assay. A 10 mg/mL stock solution of CS from shark cartilage (Millipore Sigma) was prepared in deionized water. That stock was then diluted to a final concentration of 0.5 mg/mL in bacterial supernatant and incubated anaerobically at 37°C overnight. Alcian Blue dye stock was prepared by diluting 0.5 g of Alcian Blue (VWR) in 100 mL of 18 mM H_2_SO_4_, centrifuging the solution at 10,000 × *g* for 30 min, and filtering. CS standards ranging from 2 to 0.004 mg/mL were prepared in deionized water. After the overnight incubation, 10 μL of standards or sample were added to a microcentrifuge tube. This was followed by the addition of 10 μL of sample diluent (4 M guanidine containing 0.0375% Triton X-100 in 27 mM H_2_SO_4_) and 100 μL of working Alcian Blue solution (5% dye stock in 18 mM H_2_SO_4_ + 0.25% Triton X-100). The tubes containing samples or standards were then vortexed briefly to mix and centrifuged at 10,000 × *g* for 10 min. The supernatant was then decanted, and the pellets were left to dry for at least 10 min. Finally, the pellets were dissolved in 100 μL of 8 M guanidine by vortexing. The solutions were then pipetted into a 96-well plate, and the absorbance was read at 600 nm. The generation of a standard curve allowed us to quantify the final CS concentration in each sample after the supernatant-driven degradation.

### Matrigel-based basement membrane degradation model.

We designed an additional degradation model that better captured the complexity of the basement membrane, based on a tissue penetration model described by Andrian et al. (85). Matrigel (Corning) was diluted 1:3 in cold PBS, and 100 μL were added to 0.4 μm polycarbonate trans-well plate inserts (VWR). The Transwell plates were placed in a temperature of 4°C for 30 min to let the Matrigel settle, and they were then moved to an anaerobic chamber to gel at 37°C for 24 h. The next day, the Matrigel was rehydrated in 100 μL of sterile reduced PBS for 1 h at 37°C. In the meantime, a 10 mg/mL stock of 40 kDa FITC-labeled dextran (Millipore Sigma) was prepared and later diluted in either media or supernatant at a final concentration of 0.5 mg/mL. 150 μL of the supernatant containing FITC-labeled Dextran was pipetted on top of the Matrigel, and 300 μL of PBS were added to the lower chamber. The Transwell plates were then incubated anaerobically for 24 h at 37°C. Fluorescence in the bottom chamber was measured to assess the permeability of the Matrigel layer. Because dextran is a carbohydrate that could be digested by gut bacteria, the percentage of dextran that successfully traversed the membrane was calculated in comparison to the fluorescence levels in the leftover FITC-dextran and supernatant solution after the same anaerobic incubation at 37°C.

### Preparation of human stool supernatants.

Stool microbiome samples were obtained from informed patients who provided consent during colonic irrigation procedures in accordance with the Institutional Review Board (IRB) protocols for Weill Cornell Medical College (number 1501015812) and Cornell University (number 1609006586). Ulcerative colitis was defined by clinical or endoscopic characteristics. Healthy samples were collected from 2017 to 2019. Between 0.5 to 1 mL of sample were frozen after collection and moved to storage at −80°C. To prepare the stool stocks for culture, the stool was resuspended in prereduced PBS supplemented with 0.05% l-cysteine-HCl to make a stock solution. The frozen stool stocks were inoculated at a concentration of 2% (vol/vol) in 5 mL of either BHIS or GMM (57). BHIS was selected for its ability to support the growth of *Bacteroides* species, as it was the most proteolytically active in the *in vitro* assays (Fig. 1). GMM, on the other hand, was employed because of its reported ability to support the growth of a wide diversity of bacteria compared to other media (57). Liquid cultures were grown overnight for 24 h, and the supernatant was collected after centrifugation at 7,000 × *g* for 10 min. Immediately after, the proteolytic activity of these stool culture supernatants was assessed through the ECM degradation assays described above.

### DSS-iInduced colitis mouse model.

B. fragilis, *B. theta*, and *R. gnavus* supernatants were prepared by the growing of 25 mL of culture overnight in BHIS medium, centrifugation at 7,000 rpm for 10 min, and filtering through a 0.22 μm Steriflip (EMD Millipore) filter unit. The supernatants were then frozen at −80°C in 2 mL aliquots. Aliquots of BHIS medium were also prepared.

This mouse study was performed following protocols approved by the Cornell Institutional Animal Care and Use Committee (Protocol ID number 2016-0088). 45 male SPF C57BL/6 mice (The Jackson Laboratory) at 7 weeks of age were obtained for this experiment, and they were housed individually during treatments. After 1 week of acclimatization, we started treating the mice with the supernatants via daily oral gavage (200 μL per mice). Nine mice were treated per bacterial strain, with two additional control groups receiving daily gavages of BHIS medium. On the fourth day of the treatment with the supernatant, acute ulcerative colitis was induced by exposure to 1.5% (wt/vol) dextran sulfate sodium salt (DSS, 36,000-50,000 M.Wt., MP Bio) in drinking water *ad libitum* for 10 consecutive days for all of the mice, except for one of the BHIS groups, which received normal drinking water. Fresh water with DSS was replaced every three days. Five mice per group were sacrificed at the end of the DSS treatment. Following the end of the DSS treatment, the daily gavage with supernatant continued for another 3 days until the remaining mice were sacrificed. Fecal pellets were collected daily. The mice were monitored for weight loss, food and water intake, pathological features (rectal bleeding and diarrhea), and survival. They were also inspected for visible clinical signs of pathology. The presence of diarrhea, rectal bleeding, and weight loss were separately graded on a 0 to 3 scale (Table S5) adapted from Gommeaux et al. (86). The scores were then added to calculate the disease activity index (DAI).

10.1128/mbio.02201-22.5 TABLE S5 Criteria for scoring the disease activity index (DAI). Download Table S5, DOCX file, 0.01 MB. Copyright © 2022 Porras et al. 2022 Porras et al. https://creativecommons.org/licenses/by/4.0/ This content is distributed under the terms of the Creative Commons Attribution 4.0 International license.

### Histological and immunofluorescent characterization of explanted mouse colons.

Sections (0.2 to 0.5 cm) of the terminal colon were collected after euthanasia, fixed in either formalin or methacarn for 24 h, and later placed in 70% or 100% ethanol, respectively. The tissue sections were then paraffin embedded and sectioned at the Animal Health Diagnostic Center at the Cornell University College of Veterinary Medicine, where H&E staining was also performed on formalin-fixed sections. Antigen retrieval was performed prior to immunofluorescent staining by heating the tissue sections in citric acid buffer (pH 6.0; Vector Laboratories) at 95°C for 20 min. The sections were then washed with PBS and blocked with 10% goat serum overnight. This was followed by another overnight incubation at 4°C with monoclonal antibodies against laminin (1:200; Sigma-Aldrich, L9393) and collagen IV (1:400, Abcam, ab6586) diluted in 1% goat serum in PBS. After washing with PBS, secondary goat anti-rabbit IgG Alexa Fluor antibodies (Thermo Fisher, A1108) were applied, diluted 1:500 in 1% goat serum in PBS. Finally, coverslips were mounted with ProLong Gold Antifade Mountant with DAPI (Thermo Fisher). Colorimetric and fluorescent images were obtained on an inverted Leica DMi8 microscope. The H&E images were blinded prior to histopathological scoring, and we used the method described by Bonfiglio et al. (87) to quantitatively describe the lamina propria cellularity, architectural damage, and epithelial abnormalities (Table S5).

### Quantification of lipocalin-2 in mouse stool.

We followed the protocol by Chassaing et al. to assess the lipocalin-2 levels in the mouse stool (58). Stool pellets were reconstituted in PBS containing 0.1% Tween 20 (100 mg stool/mL). This was followed by 10 min of vortexing and centrifugation at 10,000 × *g* for 10 min. The supernatant was then collected and frozen at −20°C. The LCN-2 levels were quantified later by ELISA (DY1851, R&D Systems).

### 16s rRNA gene sequencing.

We extracted genomic DNA from human stool cultures or from mouse fecal pellets using Qiagen DNeasy PowerSoil Kits, following the manufacturer’s instructions. The V4 region of the 16S rRNA gene was amplified in triplicate, following the Earth Microbiome Project protocols (88) and using barcoded 515F (89) and 806R (90) primers as well as the Platinum Hot Start PCR Master Mix (Thermo Fisher). The PCR products were cleaned using AMPure XP beads and pooled for each sample. Prior to sequencing, amplicon pools were quantified with a Quant-iT PicoGreen dsDNA Assay Kit (Invitrogen). 100 ng of amplicons from each sample were pooled prior to submission for paired-end sequencing on an Illumina MiSeq platform at the Cornell Institute of Biotechnology.

16S rRNA gene sequences were analyzed using the Quantitative Insights into Microbial Ecology (QIIME2; https://qiime2.org/) pipeline. First, we performed quality control with DADA2 (80) to remove chimeric sequences, retain unique sequence variants, and trim the forward and reverse reads. Taxonomies were assigned using QIIME2’s Naive Bayes classifier, trained with the (Greengenes Database). We then used the scipy.spatial.distance.braycurtis function to compute the Bray-Curtis distances.

### Supernatant preparation for nano LC/MS/MS.

Following bacterial culture as described above, the supernatant was collected and filtered using a 10 kDa Amicon ultra-4 centrifugal filter unit (Millipore Sigma) at 15,000 × *g* and 4°C for 15 min. The supernatant was then concentrated 10-fold in PBS containing the SIGMA*FAST* protease inhibitor (Millipore Sigma), frozen at −20°C, and submitted to the Proteomics Facility at the Cornell Institute of Biotechnology. In solution, the digestion for each sample was performed with an S-Trap micro spin column (ProtiFi, Huntington, NY, USA), following the S-Trap protocol as described previously (91, 92), with slight modifications. 30 μg of proteins in 25 μL buffer containing 50 mM TEAB (pH 8.5), 6 M urea, 2 M thiourea, and 1% SDS were reduced with 15 mM dithiothreitol (DTT) for 1 h at 34°C, alkylated with 50 mM iodoacetamide for 1 h in the dark, and then quenched with a final concentration of 25 mM DTT. After quenching, 12% phosphoric acid was added to each sample for a final concentration of 1.2%. This was followed by 1:7 dilution (vol/vol) with 90% methanol, 0.1 M TEAB (pH 8.5). Each of the resulting samples was then placed into a spin column and centrifuged at 3000 × *g* for 30 sec. Then, the samples were washed three times with 150 μL 90% methanol and 0.1 M TEAB (pH 8.5). Digestion was performed by adding 25 μL trypsin at 1:10 (wt/wt) (trypsin: proteins) in 50 mM TEAB (pH 8.5) to the top of the spin column. The spin columns were incubated overnight (16 h) at 37°C. Following the incubation, the digested peptides were eluted off the S-trap column sequentially with 40 μL each of 50 mM TEAB (pH 8.5) followed by 0.2% formic acid and 50% acetonitrile, 0.2% formic acid. Three eluates with eluted peptides were pooled and evaporated to dryness by a Speedvac SC110 (Thermo Savant, Milford, MA).

### Identification of proteins in bacterial supernatants.

The tryptic digests were reconstituted in 0.5% formic acid (FA) for a nano liquid chromatography tandem mass spectrometry (nano-LC-ESI MS/MS) analysis. The analysis was carried out using an Orbitrap Fusion Tribrid (Thermo-Fisher Scientific, San Jose, CA) mass spectrometer equipped with a Nanospray Flex Ion Source and coupled with a Dionex UltiMate 3000 RSLCnano system (Thermo, Sunnyvale, CA) (91, 93). The peptide samples (20 μL) were injected onto a PepMap C-18 RP nano trapping column (5 μm, 100 μm i.d. × 20 mm) at a 20 μL/min flow rate for rapid sample loading. The samples were then separated on a PepMap C-18 RP nano column (2 μm, 75 μm × 25 cm) at 35°C. The tryptic peptides were eluted in a 60 min gradient of 7% to 38% ACN in 0.1% formic acid at 300 nL/min, followed by a 7 min ramping to 90% ACN-0.1% FA and an 8 min hold at 90% ACN-0.1% FA. The column was re-equilibrated with 0.1% FA for 25 min, prior to the next run. The Orbitrap Fusion was operated in the positive ion mode with the spray voltage set at 1.9 kV and the source temperature set at 275°C. External calibration for the FT, IT, and quadrupole mass analyzers was performed. The data-dependent acquisition (DDA) mode was used for analysis. The instrument was operated using an FT mass analyzer during MS scan to select precursor ions followed by 3 s “Top Speed” data-dependent CID ion trap MS/MS scans at 1.6 *m/z* quadrupole isolation for the precursor peptides with multiple charged ions above a threshold ion count of 10,000 and a normalized collision energy of 30%. The MS survey scans were set at a resolving power of 120,000 (fwhm at *m/z* 200) for the mass range of *m/z* 375 to 1,575. Dynamic exclusion parameters were set at 50 s of exclusion duration with an exclusion mass width of ±10 ppm. All of the data were acquired using Xcalibur 4.4 operation software (Thermo Fisher Scientific).

The peptides were identified against the corresponding genomes that were downloaded from the NCBI RefSeq Database (Table S1). Open reading frames were predicted using Prodigal v2.6.3 (94). The resulting coding sequences were annotated by aligning them to the Carbohydrate Active Enzyme database (http://www.cazy.org/) (95) using DIAMOND BLASTP (identity ≥40%; coverage >80%; E value <1e−5) (96). Protein families were annotated on the Pfam-A 33.1 database using Hmmsearch v3.1 (97). For every ECM component, we compiled a list of the Pfams and CAZymes that were secreted by the species capable of degrading that component. We then manually inspected all of the Pfams and CAZymes to identify those that were reported to be associated with or capable of ECM degradation (Tables S3 and S4).

### Analysis of proteases and CAZymes in IBD cohorts.

We downloaded the PRISM data set (59) and removed the samples with abnormally low (less than 10^7^) reads. Low-quality reads were removed using Trimmomatic-0.3 (98). We used HUMAnN3 with the default settings to define the functional potential of the gut metagenome. As described in the previous section, we generated a list of protein families and CAZymes secreted by the bacterial species in the *in vitro* experiments that were associated with ECM degradation. We searched this curated list against the Uniref90 groups identified in the PRISM data set using DIAMOND BLASTP, requiring greater than 50% sequence identity and greater than 80% coverage. For each sample, we aggregated the abundances of the Uniref90 groups according to their corresponding protein families. The fold change differences were compared by a Mann-Whitney U-test with the false discovery rate (FDR) correction (FDR < 0.05).

### Statistical analysis.

The statistical analyses for all of the experiments were performed using GraphPad Prism v9, except for the analysis of the protease and CAZyme abundances in the IBD metagenomic cohort. For all of the strain-level experiments, the groups were compared using a one-way analysis of variance (ANOVA), followed by Tukey’s multiple-comparison test. A two-way ANOVA followed by Tukey’s multiple-comparison test was selected for the evaluation of the human clinical samples. Finally, the *in vivo* data were analyzed using a mixed-effects model that took into account repeated measures over time with the Geisser-Greenhouse correction and followed by Tukey’s multiple-comparison test. In each cases, the difference between two experimental groups was considered to be statistically significant when the *P* value was less than 0.05 after the multiple-comparison corrections.
