# Supplementary material for: Inflammatory Bowel Disease-Associated Gut Commensals Degrade Components of the Extracellular Matrix
Source: mBio. 2022 Nov 29;13(6):e02201-22. doi: 10.1128/mbio.02201-22 (PMC9765649; doi:10.1128/mbio.02201-22)
Supplement: TABLE S1 [file mbio.02201-22-s0003.docx]

**Supplementary Table 1. List of bacterial strains tested in degradation assays *in vitro*.**

| **Bacterial species** | **Strain** | **Source** | **Growth Medium** |
| --- | --- | --- | --- |
| *Akkermansia muciniphila* | Muc | DSM 22959 | PYG Medium with 0.5% mucin |
| *Bacteroides fragilis* | NCTC 9343 | ATCC 25285 | Supplemented Brain Heart Infusion Broth |
| *Bacteroides fragilis* | 2-078382-3 | ATCC 43858 | Supplemented Brain Heart Infusion Broth |
| *Bacteroides fragilis* | MPRL 1842 | DSM 9669 | Supplemented Brain Heart Infusion Broth |
| *Bacteroides thetaiotaomicron* | VPI 5482 | DSM 2079 | Supplemented Brain Heart Infusion Broth |
| *Bacteroides ovatus* | NCTC 11153 | ATCC 8483 | Supplemented Brain Heart Infusion Broth |
| *Bacteroides vulgatus* | NCTC 11154 | ATCC 8482 | Supplemented Brain Heart Infusion Broth |
| *Bifidobacterium longum* | S12 | ATCC 15697 | ATCC Medium 2107: Modified Reinforced Clostridial |
| *Enterococcus faecalis* | Tissier | DSM 20478 | Trypticase Soy Yeast Extract Medium |
| *Escherichia coli* | Nissle 1917 |  | Luria Broth |
| *Lactobacillus gasseri* | F 164 | DSM 20077 | MRS Medium |
| *Lactobacillus reuteri* | MM4-1A | ATCC PTA-6475 | MRS Medium |
| *Ruminococcus gnavus* | H2_28 | DSM 108212 | PYG Medium |
| *Prevotella copri* | CB7 | DSM 18205 | BBL^TM^ Schaedler Broth |
| *Prevotella copri* | S6-G7 isolate | Isolated from Fijian donor | BBL^TM^ Schaedler Broth |
| *Prevotella copri* | S6-C12 isolate | Isolated from Fijian donor | BBL^TM^ Schaedler Broth |
| *Prevotella copri* | S6-D12 isolate | Isolated from Fijian donor | BBL^TM^ Schaedler Broth |
