# Supplementary material for: Inflammatory Bowel Disease-Associated Gut Commensals Degrade Components of the Extracellular Matrix
Source: mBio. 2022 Nov 29;13(6):e02201-22. doi: 10.1128/mbio.02201-22 (PMC9765649; doi:10.1128/mbio.02201-22)
Supplement: TABLE S2 [file mbio.02201-22-s0004.docx]

**Supplementary Table 2. Metadata of healthy and UC patients participating in this study.**

| **Healthy controls** | | | | **Ulcerative colitis** | | | |
| --- | --- | --- | --- | --- | --- | --- | --- |
| **Sample ID** | **Patient ID** | **Age** | **Gender** | **Sample ID** | **Patient ID** | **Age** | **Gender** |
| 1B | 697 | 37 | Male | 1A | 304 | 37 | Male |
| 2B | 832 | 22 | Male | 2A | 373 | 24 | Male |
| 3B | 268 | 40 | Female | 3A | 479 | 40 | Male |
| 4B | 693 | 57 | Male | 4A | 567 | 57 | Female |
| 5B | 1020 | 65 | Female | 5A | 598 | 65 | Female |
| 6B | 1143 | 31 | Female | 7A | 232 | 29 | Male |
| 7B | 281 | 29 | Male | 8A | 468 | 24 | Female |
| 8B | 660 | 27 | Female | 9A | 739 | 56 | Female |
| 9B | 1073 | 56 | Female | 10A | 778 | 42 | Male |
| 10B | 872 | 43 | Male |  |  |  |  |
