# Supplementary material for: Inflammatory Bowel Disease-Associated Gut Commensals Degrade Components of the Extracellular Matrix
Source: mBio. 2022 Nov 29;13(6):e02201-22. doi: 10.1128/mbio.02201-22 (PMC9765649; doi:10.1128/mbio.02201-22)
Supplement: TABLE S3 [file mbio.02201-22-s0005.docx]

**Supplementary Table 3. Protein families (Pfams) associated with ECM degradation secreted by bacterial strains *in vitro*.** List of Pfams secreted by bacterial species *in vitro* with reported roles involved in the degradation of ECM components. The “ECM Component” column indicates that Pfam was identified in the supernatant of bacteria capable of degrading that particular component.

| **Pfam ID** | **Name** | **ECM Component** |
| --- | --- | --- |
| PF01120.19 | Alpha-L-fucosidase | Laminin, collagen |
| PF02127.17 | Aminopeptidase I zinc metalloprotease (M18) | Laminin |
| PF01400.26 | Astacin (Peptidase family M12A) | Laminin |
| PF00648.23 | Calpain family cysteine protease | Fibronectin, laminin |
| PF13620.8 | Carboxypeptidase regulatory-like domain | Laminin |
| PF08669.13 | Glycine cleavage T-protein C-terminal barrel domain | Laminin, collagen |
| PF03065.17 | Glycosyl hydrolase family 57 | Fibronectin |
| PF14509.8 | Glycosyl-hydrolase 97 C-terminal, oligomerisation | Laminin |
| PF17829.3 | Gylcosyl hydrolase family 115 C-terminal domain | Laminin |
| PF02275.20 | Linear amide C-N hydrolases, choloylglycine hydrolase family | Laminin |
| PF13582.8 | Metallo-peptidase family M12B Reprolysin-like | Fibronectin, laminin, collagen |
| PF00112.25 | Papain family cysteine protease | Fibronectin, laminin |
| PF01640.19 | Peptidase C10 family | Fibronectin, laminin |
| PF01364.20 | Peptidase family C25 | Fibronectin, laminin |
| PF03577.17 | Peptidase family C69 | Fibronectin |
| PF01551.24 | Peptidase family M23 | Laminin |
| PF03571.17 | Peptidase family M49 | Laminin |
| PF01136.21 | Peptidase family U32 | Laminin |
| PF00639.23 | PPIC-type PPIASE domain | Collagen |
| PF16141.7 | Putative glycoside hydrolase Family 18, chitinase_18 | Laminin |
| PF00082.24 | Subtilase family | Laminin |
| PF00089.28 | Trypsin | Fibronectin, laminin |
| PF13365.8 | Trypsin-like peptidase domain | Laminin, collagen, fibronectin |
