# Supplementary material for: Inflammatory Bowel Disease-Associated Gut Commensals Degrade Components of the Extracellular Matrix
Source: mBio. 2022 Nov 29;13(6):e02201-22. doi: 10.1128/mbio.02201-22 (PMC9765649; doi:10.1128/mbio.02201-22)
Supplement: TABLE S4 [file mbio.02201-22-s0006.docx]

**Supplementary Table 4. CAZymes associated with glycosaminoglycan degradation secreted by bacterial strains *in vitro*.** List of CAZymes secreted by bacterial species *in vitro* with reported roles involved in the degradation of glycosaminoglycans (in this case, HA and CS).

| **CAZy ID** | **Functions** |
| --- | --- |
| CE5 | acetyl xylan esterase |
| GH57 | alpha-amylase, alpha-galactosidase |
| GH13 | alpha-amylase, pullulanase |
| GH31 | alpha-glucosidase, alpha-galactosidase, alpha-mannosidase, alpha-xylosidase |
| GH29 | alpha-L-fucosidase; alpha-1,3/1,4-L-fucosidase |
| GH38 | alpha-mannosidase |
| GH109 | alpha-N-acetylgalactosaminidase |
| GH2 | beta-galactosidase, beta-mannosidase |
| GH35 | beta-galactosidase, exo-beta-glucosaminidase |
| GH3 | beta-glucosidase, xylan 1,4-beta-xylosidase, beta-glucosylceramidase |
| GH20 | beta-hexosaminidase, lacto-N-biosidase, beta-1,6-N-acetylglucosaminidase |
| GH120 | beta-xylosidase |
| GH43 | beta-xylosidase, alpha-L-arabinofuranosidase, xylanase |
| GH18 | chitinase, lysozyme |
| GH101 | endo-alpha-N-acetylgalactosaminidase |
| GH51 | endoglucanase, endo-beta-1,4-xylanase, beta-xylosidase |
| GH125 | exo-alpha-1,6-mannosidase |
| GH97 | glucoamylase, alpha-glucosidase, alpha-galactosidase |
| GH0 | glycoside hydrolases not yet assigned to a family. |
| GH92 | mannosyl-oligosaccharide alpha-1,2-mannosidase |
| GH84 | N-acetyl beta-glucosaminidase, hyaluronidase |
| CE9 | N-acetylglucosamine 6-phosphate deacetylase |
