# Supplementary material for: Inflammatory Bowel Disease-Associated Gut Commensals Degrade Components of the Extracellular Matrix
Source: mBio. 2022 Nov 29;13(6):e02201-22. doi: 10.1128/mbio.02201-22 (PMC9765649; doi:10.1128/mbio.02201-22)
Supplement: TABLE S5 [file mbio.02201-22-s0007.docx]

**Supplementary Table 5. Criteria for scoring the disease activity index (DAI).**

| **Score** | **Weight lost (% of initial)** | **Stool consistency** | **Rectal bleeding** |
| --- | --- | --- | --- |
| 0 | <1 | Normally formed pellets | None |
| 1 | 1-4.99 | Soft pellets not adhering to the anus | Small spots of blood in stool; dry anal region |
| 2 | 5-10 | Very soft pellets adhering to the anus | Large spots of blood in stool; blood appears through anal orifice |
| 3 | >10 | Liquid stool on long streams; wet anus | Deep red stool; blood spreads largely around the anus |
